# In Hyperthermia Increased ERK and WNT Signaling Suppress Colorectal Cancer Cell Growth

**DOI:** 10.3390/cancers8050049

**Published:** 2016-05-14

**Authors:** Michael Bordonaro, Senji Shirasawa, Darina L. Lazarova

**Affiliations:** 1 Department of Basic Sciences, The Commonwealth Medical College, Scranton, PA 18509, USA; mbordonaro@tcmc.edu; 2 Department of Cell Biology, Fukuoka University, Fukuoka 814-0180, Japan; sshirasa@fukuoka-u.ac.jp

**Keywords:** Hyperthermia, colorectal cancer, KRAS, ERK, WNT

## Abstract

Although neoplastic cells exhibit relatively higher sensitivity to hyperthermia than normal cells, hyperthermia has had variable success as an anti-cancer therapy. This variable outcome might be due to the fact that cancer cells themselves have differential degrees of sensitivity to high temperature. We hypothesized that the varying sensitivity of colorectal cancer (CRC) cells to hyperthermia depends upon the differential induction of survival pathways. Screening of such pathways revealed that Extracellular Signal-Regulated Kinase (ERK) signaling is augmented by hyperthermia, and the extent of this modulation correlates with the mutation status of V-Ki-ras2 Kirsten rat sarcoma viral oncogene homolog (*KRAS*). Through clonal growth assays, apoptotic analyses and transcription reporter assays of CRC cells that differ only in *KRAS* mutation status we established that mutant *KRAS* cells are more sensitive to hyperthermia, as they exhibit sustained ERK signaling hyperactivation and increased Wingless/Integrated (WNT)/beta-catenin signaling. We propose that whereas increased levels of WNT and ERK signaling and a positive feedback between the two pathways is a major obstacle in anti-cancer therapy today, under hyperthermia the hyperinduction of the pathways and their positive crosstalk contribute to CRC cell death. Ascertaining the causative association between types of mutations and hyperthermia sensitivity may allow for a mutation profile-guided application of hyperthermia as an anti-cancer therapy. Since *KRAS* and WNT signaling mutations are prevalent in CRC, our results suggest that hyperthermia-based therapy might benefit a significant number, but not all, CRC patients.

## 1. Introduction

The association between acute infections and cancer is supported by experimental and epidemiological data [1,2]. In 1874 in England, Dr. Campbell De Morgan presented evidence that in some cases cancer regresses after infections such as tuberculosis [3]. In 1890 in the U.S., during a review of records in the New York Memorial Hospital, Dr. William Coley found a case in which erysipelas, an acute Streptococcal infection, cured a cancer patient [4]. A year later, Coley replicated this outcome by utilizing *Streptococcus pyogenes* and inducing erysipelas in a patient with sarcoma [5]. Subsequently, Coley switched to a heat-inactivated mixture of bacteria, and increased the dosage until a fever of 39 °C or higher was developed by his cancer patients [4,6,7]. Most of Coley’s patients had late stage cancers that did not respond to conventional treatments and yet, retrospective analyses report five-year survival for more than 44% of the patients [7]. In the 1960s, the Food and Drug Administration stopped the use of Coley’s treatment in the U.S. A later unsuccessful attempt to replicate Coley’s therapy applied a mixed bacterial vaccine (Vaccineurin); however, the treatment did not aim at achieving fever, despite the knowledge that the curative effect of acute infections is likely initiated by fever [8]. The significance of developing high body temperature was confirmed in a more recent clinical trial in Germany with a bacterial vaccine [9]. Epidemiological data have also supported an inverse association between acute infections accompanied by high fever and cancer incidence. For example, individuals with a history of three or more infections with fever above 38.5 °C have a 40% lower risk of melanoma [10], and the anamnesis of cancer patients compared to the medical history of infectious diseases in cancer-free patients has been confirmed [11].

In contrast to the inverse association between acute infections and cancer, chronic inflammations increase the risk of cancer [1]. A significant difference between the two conditions is that acute inflammations lead to high fever compared to chronic inflammations [2], and fever might be the critical anti-cancer factor, since neoplastic cells are more sensitive to higher temperatures [8]. Furthermore, the release of internal neoantigens from hyperthermia-killed neoplastic cells may elicit anti-cancer immune response [11]. Therefore, the therapeutic response to hyperthermia likely consists of two steps: A signaling response at the cancer cell level, and an immune response at the level of the organism [2,11]. We have focused on the mechanisms of the first step, since cell signaling differences defined by the cancer mutation profile may explain the differential sensitivity of cancers to hyperthermia.

Based upon our *in vitro* results, we propose that a subset of colorectal cancers (CRCs) with mutations in *V-Ki-ras2 Kirsten rat sarcoma viral oncogene homolog (KRAS)* and Wingless/Integrated (WNT)/beta-catenin signaling might be most sensitive to the effects of hyperthermia as an anti-cancer therapy. The three most frequently mutated genes in microsatellite stable CRC, the most common form of CRC, are *Adenomatous Polyposis Coli* (*APC*), *KRAS*, and *Tumor protein 53* (*TP53*) [12]. Previous research has revealed that mutations in *TP53* increase the resistance of cancer cells to hyperthermia [13,14]. Therefore, a CRC mutation profile of a wild type *TP53*; mutant *KRAS*, mutant *APC* (or *Cadherin-Associated Protein beta1* gene, *CTNNB1*) is predicted to be most sensitive to hyperthermia-induced apoptosis. This might be a common mutation phenotype, since the co-occurrence of *KRAS* and *APC* mutations is statistically significant (*p* = 0.004, log of odds ratio 0.903); whereas, the co-occurrence of a *TP53* mutation with an *APC* or *KRAS* mutation is either not statistically significant (*p* = 0.385, log of odds ratio 0.134) and mutually exclusive (*p* = 0.453, log of odds ratio −0.069), respectively (http://www.cbioportal.org, The Cancer Genome Atlas (TCGA) provisional database analyses, accessed on 14 August 2015). Although focusing on mutations in three genes might be perceived as simplistic, recent sequencing analyses have revealed that the average number of driver gene mutations in CRC is three to five [15,16]. Missense *KRAS* mutations are present in 40%–45% of the CRC patients and WNT/beta-catenin activity is deregulated via mutations in more than 80% of CRC patients [17,18,19,20,21,22]; therefore, findings from our studies may impact the therapeutic options for a considerable number of patients with this malignancy.

## 2. Results

### 2.1. CRC Cells with a Mutant KRAS Are More Sensitive to Hyperthermia Than CRC Cells with a Wild Type KRAS

**Figure 1:**
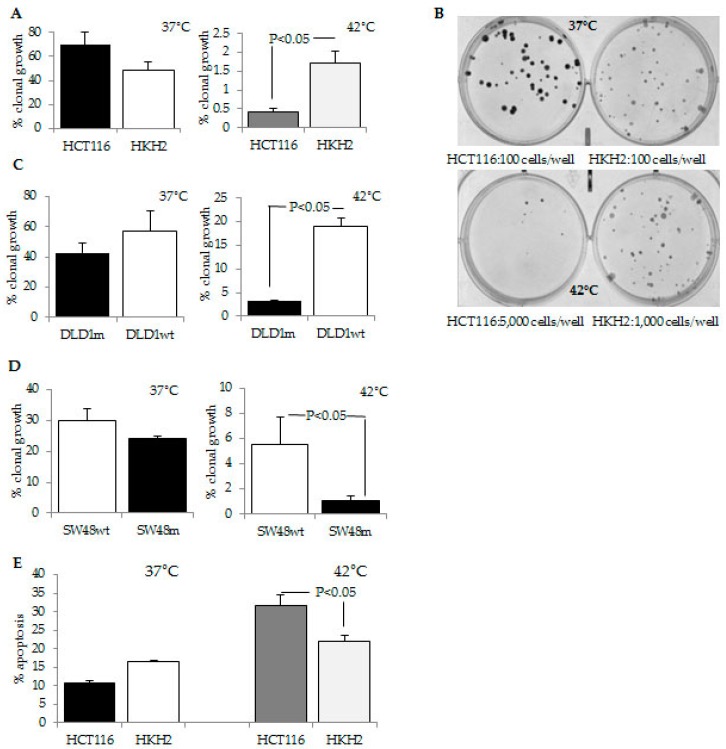
Differential hyperthermia response of mutant and wild type *KRAS* CRC cells. (**A**) Clonal growth assays. Cells were plated at 100 per well in six-well plates and incubated at 37 °C for two weeks, or at 5000 cells/well (HCT-116) and 1,000 cells/well (HKH2), incubated at 42 °C for 24 h, and transferred to 37 °C for two weeks. The percent clonal growth is the ratio of number of colonies to number of all plated cells, multiplied by 100; (**B**) Representative results from clonal growth analyses described in (A); (**C**) Isogenic DLD-1m cells (with a mutant *KRAS,*G12D) and DLD-1wt (DLD-1 cells with a wild type *KRAS*) were exposed to 37 °C or 42 °C for 24 h, and clonal growth was measured as described in (A); (**D**) Isogenic SW48wt cells (with a wild type *KRAS*) and SW48m (with a *KRAS* mutation G12D) were assayed for clonal growth as described in (A). All clonal growth assays were performed with triplicate samples per treatment conditions; three to five assays were performed at each temperature; (**E**) Cells were exposed to 37 °C or 42 °C for 24 h. All cells (floating and attached) were harvested and stained for apoptotic and necrotic markers with R-phycoerythrin (PE)––Annexin V Apoptosis Detection Kit I (BD Biosciences, San Jose, CA, USA). Four experiments with triplicate samples per treatment were performed.

We posited that the sensitivity of colorectal cancer (CRC) cells to hyperthermia depends upon the modulation of oncogene-deregulated cell survival signaling. Screening of several major proliferative pathways in HCT-116 CRC cells exposed to hyperthermia revealed that exposure of the cells to 42 °C modulates ERK activity. Since this signaling pathway is dependent upon the mutational status of *KRAS*, a gene that is commonly mutated in CRC, we ascertained the role of KRAS in the sensitivity of cancer cells to hyperthermia. For this purpose, we used isogenic CRC cells with a different *KRAS* mutation status: HCT-116 cells with a *KRAS* activating mutation at codon 13, and the isogenic cells HKH2 cells, in which the mutant *KRAS* allele is disrupted via homologous recombination [23]; DLD-1 parental cells with a *KRAS* mutation (G13D) and DLD-1 cells, in which the mutant *KRAS* allele is knocked-out (Horizon Discovery Group, Cambridge, UK); SW48 parental cells with a wild type *KRAS* gene and the isogenic SW48 cells with a knocked-in *KRAS* mutant allele G12D (Horizon Discovery Group). We utilized incubations at 42 °C, as this temperature is achievable in a whole-body hyperthermia procedure and it corresponds to a fever-range temperature [24]. At 37 °C, HCT-116 and HKH2 cells exhibited 69.6% ± 10.7% and 48.4% ± 6.6% clonal growth, respectively (*p* > 0.05), and at 42 °C, HCT-116 and HKH2 cells exhibited 0.42% ± 0.1% and 1.73% ± 0.3% clonal growth, respectively (*p* < 0.05, Figure 1). At 42 °C, DLD-1 parental cells with a *KRAS* mutation (G13D) also exhibited higher hyperthermia sensitivity (3.2% ± 0.15 % clonal growth) than DLD-1 cells, in which the mutant *KRAS* allele was knocked-out (18.97% ± 1.75%, *p* < 0.05, Figure 1). Similarly, at 42 °C SW48 parental cells that are wild type *KRAS* cells, exhibited lower hyperthermia sensitivity (5.55% ± 2.1%) than the isogenic SW48 cells with a knocked-in *KRAS* mutant allele G12D (1.06% ± 0.38%, *p* < 0.05, Figure 1). Further studies were carried out with the most hyperthermia-sensitive cells, HCT-116, and their isogenic counterparts, HKH2 cells. Apoptotic analyses revealed that incubations at 42 °C resulted in a three-fold increase in apoptosis in HCT-116 cells: from 10.8% ± 0.6% apoptosis at 37 °C to 31.8% ± 2.6% apoptosis at 42 °C, *p* < 0.05 (Figure 1). The same treatment of HKH2 cells led to a 1.3-fold increase in apoptosis: at 37 °C, the cells exhibited 16.4% ± 0.3% apoptosis, and these levels increased to 22.1% ± 1.5% at 42 °C, *p* < 0.05 (Figure 1).

### 2.2. CRC Cells with Differential Hyperthermia Sensitivity Exhibit Differential Temporal Activation of ERK Signaling

**Figure 2:**
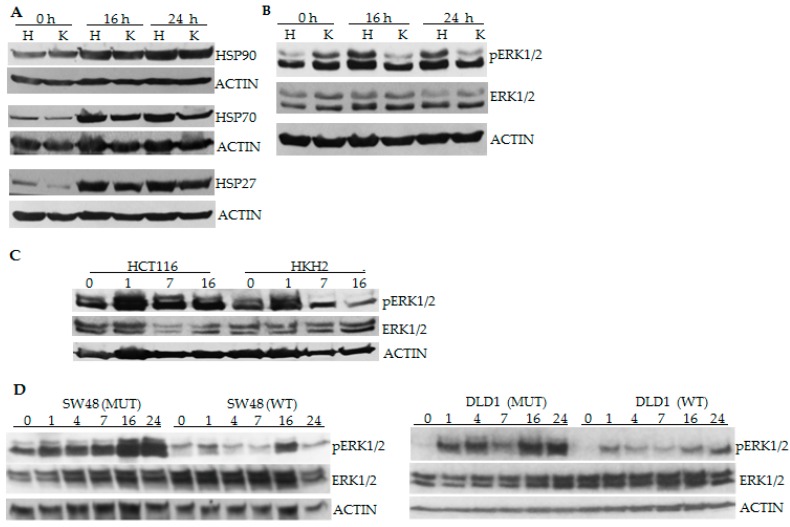
The induction of ERK1/2 by hyperthermia exhibits a different temporal pattern in CRC cells with a wild type and mutant *KRAS*. (**A**–**C**) HCT-116 (H) and HKH2 (K) cells were exposed to 42 °C for 0, 1, 7, 16 or 24 h. Total cell lysates were analyzed by Western blotting, and probed for heat shock proteins (HSP), total ERK1/2, phosphorylated (p) ERK1/2 and ACTIN; (**D**) SW48 and DLD-1 pairs of isogenic cell lines with a wild type (WT) or mutant (MUT) *KRAS* were exposed to 42 °C for 0, 1, 7, 16 or 24 h and total cell lysates were analyzed as in (A).

To identify the molecular mechanisms contributing to the higher hyperthermia sensitivity of mutant *KRAS* CRC cells, we determined whether the levels of three major heat-shock proteins differ between the isogenic cells. Differentially increased levels of heat-shock proteins could account for differential thermosensitivity of the cells. Western blot analyses ascertained similar increase in the levels of HSP27, HSP70, and HSP90 in HCT-116 and HKH2 cells under hyperthermia (Figure 2A). Since the two cell types differ in their *KRAS* mutation status, we also compared the relative levels of the downstream ERK1/2 signaling at 16 and 24 h of hyperthermia. Unlike HKH2 cells, the exposure of HCT-116 cells to 42 °C resulted in increased phosphorylation of ERK1 and ERK2 at 16 and 24 h of exposure (Figure 2B). Analyses of cells exposed to hyperthermia for shorter periods of time revealed that HKH2 cells also activate ERK1/2; however, the increase in pERK1/2 levels is transient compared to this in HCT-116 cells (Figure 2C). Similar results were obtained with the two additional isogenic pairs of cells (SW48 and DLD-1). However, in these cells, the increase of pERK1/2 levels had a pulsatile pattern, and this pulsatility was more pronounced in cells with wild type *KRAS* gene (Figure 2D).

**Figure 3:**
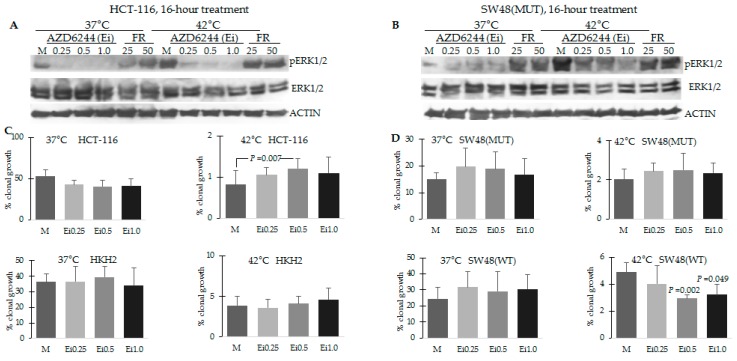
Hyperthermia-induced ERK activity has anti-proliferative effects in mutant *KRAS* CRC cells. (**A**,**B**) Cells were exposed for 16 h to 37 °C or 42 °C, and mock treatment (M), AZD6244 at 0.25, 0.5 and 1.0 μM, or FR180204 (FR) at 25 and 50 μM. Total cell lysates were analyzed by Western blotting, and probed for total ERK1/2, phosphorylated (p) ERK1/2 and ACTIN as described in Figure 2; (**C**,**D**) Clonal growth assays as described in Figure 1 were performed with cells exposed to AZD6244 (Ei) at 0.25, 0.5 and 1.0 μM.

Depending upon the cellular context and conditions, ERK activity may contribute to cell proliferation, or differentiation and apoptosis. To distinguish between these roles of ERK signaling under the conditions of hyperthermia, we decided to inhibit the pathway with a MEK1/2 inhibitor (AZD6244) or an ERK inhibitor (FR180204) and evaluate the effect of suppressed ERK activity on clonal cell growth. First, we ascertained the ability of the two inhibitors to modulate ERK activation in the cells with mutant *KRAS,* as these cells exhibited the highest activation of the pathway under hyperthermia. Under the conditions used, AZD6244, but not FR180204, inhibited the increased pERK1/2 levels in HCT-116 and to a lesser extent, in the mutant *KRAS* SW48 cells (Figure 3A). Therefore, we performed clonal growth assays only in the presence and absence of increasing concentrations of AZD6244. At 42 °C, mock treatment of HCT-116 cells resulted in 0.82% ± 0.33 % clonal growth, and increasing concentrations of AZD6244 (0.25, 0.5, and 1.0 μM) resulted in increased ability of the cells to survive hyperthermia and form colonies: 1.05% ± 0.18 %, 1.21% ± 0.24 % (*p* = 0.007), and 1.1% ± 0.38 %, respectively (Figure 3C). At 42 °C, mock treatment of HKH-2 cells resulted in 3.91% ± 1.10 % clonal growth, and the exposure to increasing concentrations of AZD6244 did not result in statistically higher levels of clonal growth (3.61% ± 1.06 %, 4.12% ± 0.96 %, and 4.64% ± 1.43 % clonal growth, respectively, Figure 3C). Similarly to mutant *KRAS* HCT-116 cells, mutant *KRAS* SW48 cells increased their ability for clonal growth after hyperthermia when exposed to AZD6244; however, the changes in percent clonal growth were not statistically significant (mock-treated *KRAS* mutant SW48 cells exhibited 2.02% ± 0.55 % clonal growth, and exposure to increasing concentrations of AZD6244 resulted in 2.44% ± 0.40%, 2.50% ± 0.87%, and 2.35% ± 0.51% of clonal growth, Figure 3D). The same treatment of wild type *KRAS* SW48 cells resulted in a statistically significant decreased ability to form colonies after exposures to 42 °C: mock treated cells exhibited 4.89% ± 0.69% clonal growth, and the addition of increasing concentrations of AZD6244 led to decreased ability for colony formation: 4.03% ± 1.32%, 2.95% ± 0.25% (*p* = 0.002), and 3.21% ± 1.18% (*p* = 0.049), Figure 3D.

### 2.3. Differential Temporal Activation of ERK Signaling Correlates with Differential Activation of Epidermal Growth Factor Receptor (EGFR) in CRC Cells with Wild Type and Mutant *KRAS*

**Figure 4:**
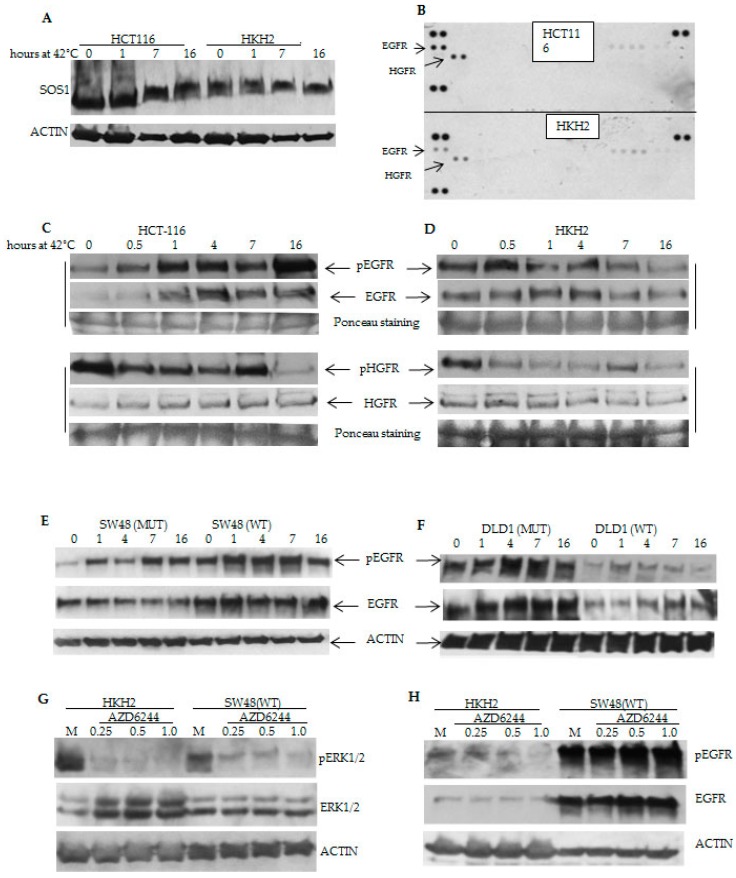
Hyperthermia modulates differentially receptor tyrosine kinase activity in *KRAS* isogenic cells. (**A**–**H**) Representative Western blot analyses with total cell lysates after incubations at 42 °C for up to 16 h (except for (**G**) and (**H**), when cells were exposed to treatments for 16 h). Antibodies are described in Materials and Methods. (A) The phosphorylation of SOS1 was detected by its delayed electrophoretic mobility on SDS-PAGE gel [25,26]; (**B**) Analyses of the activation of RTKs after 16-hour incubations at 42 °C were performed with the Proteome Profiler human phospho-RTK array (R&D Systems).

We posited that the temporal differences in hyperthermia-induced ERK signaling (Figure 2B–D) are due not only to the different *KRAS* mutation status, but also to the differential activation of receptor tyrosine kinases (RTKs). The dynamic changes in the phosphorylation status of Son of Sevenless (SOS)1, the guanine nucleotide exchange factor involved in the activation of RAS by RTKs, supported our hypothesis. The phosphorylation of SOS1 on multiple amino acid residues is usually induced by growth factor stimulation [25,26]. Our analyses revealed that in HCT-116 cells, the most sensitive to hyperthermia cells, at 42 °C and for at least up to 16 h, SOS1 was increasingly phosphorylated; the same treatment, however, decreased the phosphorylated SOS1 levels in HKH2 cells (Figure 4A). Next, we ascertained the possibility of RTK activation under hyperthermia by utilizing a Proteome Profiler human phospho-RTK array (R&D Systems, Inc., Minneapolis, MN, USA). The phospho-RTK array analyses revealed higher levels of phosphorylated EGFR and Hepatocyte Growth Factor Receptor (HGFR) in HCT-116 cells compared to HKH2 cells (Figure 4B). Western blot analyses revealed that the temporal phosphorylation changes in SOS1 corresponded to the increasing EGFR activation in HCT-116 cells, and the decreasing EGFR activation in HKH2 cells at 42 °C (Figure 4C,D). In contrast, the temporal pattern of HGFR activation was similar in the two cell types (Figure 4C,D).

Analyses with the isogenic SW48 and DLD-1 cells revealed similar increase in EGFR activation in the *KRAS* mutant cells, and decreasing levels of pEGFR in wild type *KRAS* DLD-1 cells, but not in wild type *KRAS* SW48 cells (Figure 4E,F). Therefore, we asked whether the lack of decrease in pEGFR and total EGFR levels in wild type *KRAS* SW48 cells under hyperthermia may explain why these cells responded differently from wild type *KRAS* HKH2 cells to hyperthermia combined with suppression of ERK activity (Figure 3C,D). The analyses revealed that when exposed to hyperthermia and suppression of ERK1/2 activation, the two wild type *KRAS* cell lines HKH2 and SW48 exhibit differential response: HKH2 cells decrease the levels of pEGFR; whereas, SW48 cells increase these levels (Figure 4H).

### 2.4. The Response to Hyperthermia Results in Increased WNT/beta-Catenin Activity

**Figure 5:**
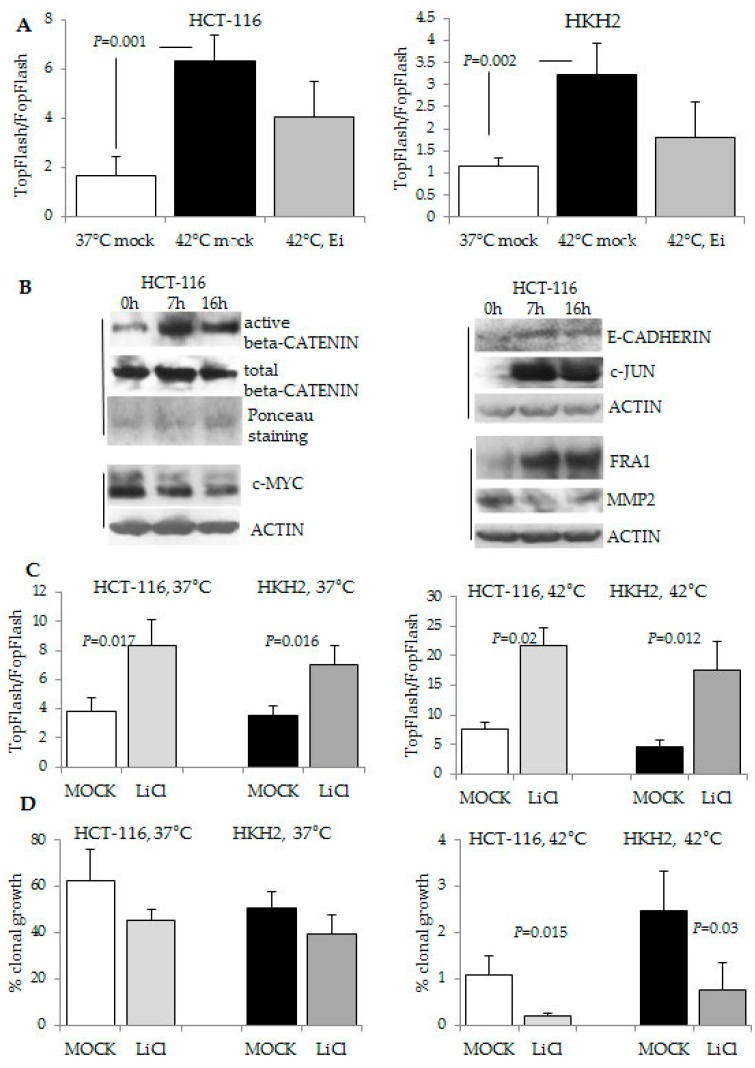
Modulation of WNT/beta-catenin activity impacts the response of CRC cells to hyperthermia. (**A**,**C**) WNT/beta-catenin transcriptional activity was measured with the luciferase reporter pair TOP-Flash and FOP-Flash. Treatments (16 h) with lithium chloride were performed at 20 mM, or with AZD6244 (Ei) at 0.5 μM. The standard deviations are based upon three experiments with duplicate samples per treatment and condition (temperature) were performed; (**B**) Representative Western blot analyses with nuclear or total cell lysates of HCT-116 cells exposed to 42 °C for 0, 7, or 16 h. Active and total beta-catenin were detected in nuclear lysates and equal loading was ascertained by Ponceau staining. Additional markers of WNT/beta-catenin activity were analyzed with total cell lysates. Vertical lines indicate same Western blot analyses; (**D**) Clonal growth assays as described in Figure 1 were performed with 20 mM lithium chloride. Standard deviation was calculated based upon three independent experiments with triplicate samples.

To understand why HCT-116 cells were the cells with the highest sensitivity to hyperthermia (Figure 1), we conducted further analyses. Whereas HCT-116 cells and their isogenic counterparts HKH2 cells differ in their *KRAS* mutation status, both cell types are heterozygous for a WNT signaling-activating mutation in *CTNNB1*^WT/Δ45^ that results in a 3-base pair deletion eliminating the serine residue at codon 45 [27]. Despite the identical *CTNNB1* mutation status, we established that mutant *KRAS* HCT-116 cells exhibit higher WNT/beta-catenin transcriptional activity levels than wild type *KRAS* HKH2 cells at 42 °C (Figure 5A). Consistent with reports that oncogenic KRAS and the resulting activation of the downstream ERK signaling enhance WNT/beta-catenin activity [28], we observed that the combined exposure of the cells to hyperthermia and a MEK1/2 inhibitor decreased the induction of WNT/beta-catenin transcriptional activity compared to hyperthermia treatment alone (Figure 5A). The increased by hyperthermia WNT/beta-catenin transcriptional activity was also ascertained through Western blot analyses that revealed that hyperthermia increases the levels of active (dephosphorylated at Ser37 or Thr41) beta-catenin; however, it has a variable effect on several direct gene targets of WNT/beta-catenin transcriptional activity (*i.e.*, at the protein level, E-CADHERIN, FRA1, and c-JUN increased; whereas, c-MYC and MMP2 protein levels decreased, Figure 5B). Considering that the combined exposure to hyperthermia and a MEK1/2 inhibitor decreased the anti-growth effect of hyperthermia on HCT-116 cells (Figure 3C), we reasoned that increasing WNT/beta-catenin transcriptional activity may augment the anti-growth effect of hyperthermia. In agreement with this hypothesis, the combined exposure of HCT-116 cells to hyperthermia and lithium chloride, a glycogen synthase kinase 3-beta inhibitor that increases WNT/beta-catenin transcriptional activity (Figure 5C), resulted in a statistically significant decrease in the ability of the cells for clonal growth (Figure 5D).

**Figure 6:**
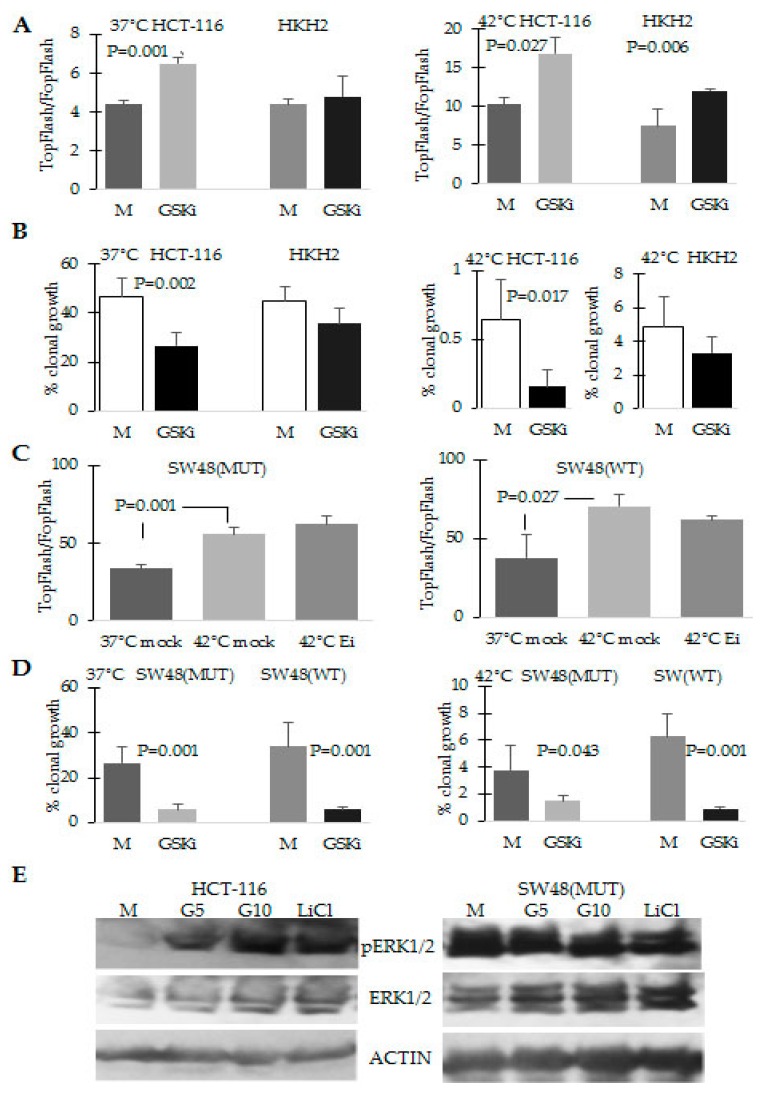
Increase of WNT/beta-catenin activity in HCT-116 cells results in higher levels of ERK1/2 activation and decreased ability for clonal growth at 42 °C. (**A**,**C**) WNT/beta-catenin transcriptional activity was measured as in Figure 5 after exposure of the cells to mock treatment (M), 5 μM of the GSK-beta kinase inhibitor (GSKi) TWS119, or 0.5 μM AZD6244 (Ei) for 16 h. Three experiments with duplicate samples per treatment and condition (temperature) were performed; (**B**,**D**) Clonal growth analyses were performed as in Figure 1 by exposing the cells for 24 h to mock treatment or 5 μM TWS119 (GSKi); (**E**) Representative Western blot analyses with total cell lysates of HCT-116 cells exposed for 16 h to 42 °C and 5 or 10 μM TWS119 (GSKi) and 20 mM lithium chloride.

The same effect of decreased ability for clonal growth under hyperthermia was observed when the cells were exposed to TWS119, another glycogen synthase kinase 3-beta inhibitor (Figure 6A); however, the decrease in clonal growth ability was statistically significant only in mutant *KRAS* HCT-116 cells (Figure 6B). Consistent with the idea that in HCT-116 cells, ERK and WNT/beta-catenin signaling amplify each other under the conditions of hyperthermia, the exposure of HCT-116 cells to the two inhibitors of synthase kinase 3-beta resulted in increased levels of ERK1/2 activation (Figure 6E).

Analyses with the pair of SW48 isogenic cell lines confirmed that increasing WNT/beta-catenin activity in combination with hyperthermia results in decreased ability for clonal growth (Figure 6D); however, the exposure of the cells to the MEK1/2 inhibitor AZD6244 did not result in decreased WNT/beta-catenin transcriptional activity (Figure 6C). Furthermore, whereas exposure of HCT-116 cells to inhibitors of synthase kinase 3-beta increased the activation of ERK1/2, the same exposure of mutant *KRAS* SW48 cells did not modulate ERK activation (Figure 6E).

### 2.5. Combination Treatment of Hyperthermia and Propolis Is Effective in Suppressing CRC Cell Growth

**Figure 7:**
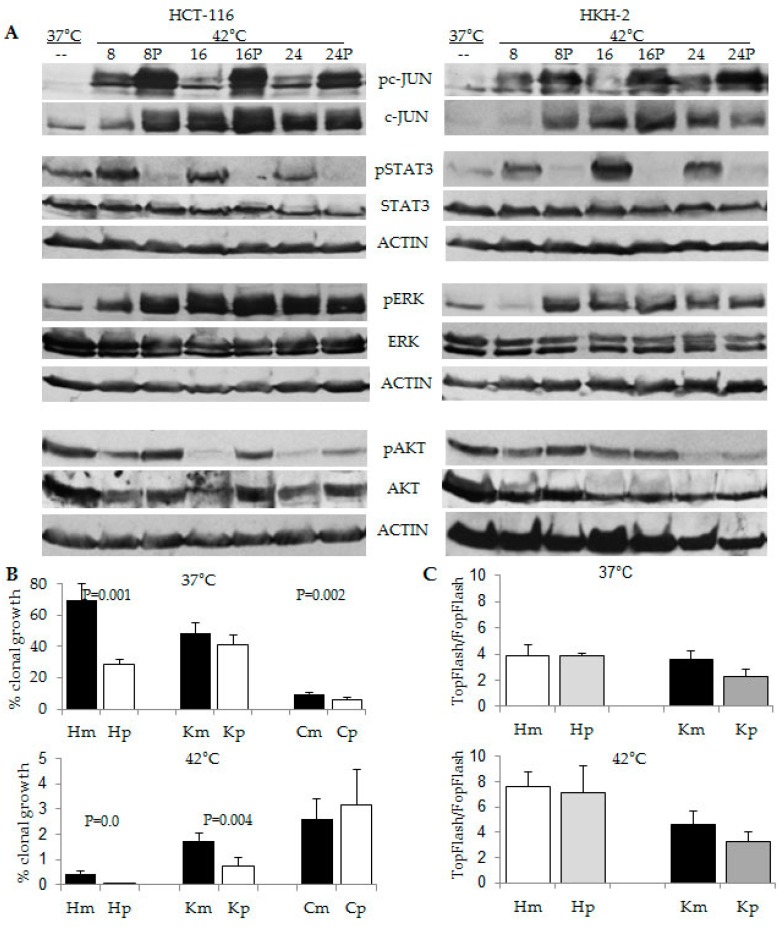
Combined effect of hyperthermia and propolis on CRC cells. (**A**) Representative Western blot analyses with total cell lysates after exposure of HCT-116 and HKH2 cells to 37 °C or 42 °C, for 8, 16, or 24 h in absence or presence of 100 μg/ml propolis (P); (**B**) Clonal growth assays of HCT-116 (H), HKH2 (K) and CDC841CoN (C) cells were performed as described in the legend of Figure 1. Cells were exposed simultaneously for 24 h to 37 °C or 42 °C and to mock (m) treatment or propolis (p) at 100 μg/mL. Results are the mean from minimum of four clonal growth assays with triplicate samples per treatment. Only statistically significant different values are indicated; (**C**) WNT/beta-catenin transcriptional activity was measured with the luciferase reporter pair TOP-Flash and FOP-Flash as described in Materials and Methods. Mock and propolis treatment were performed for 16 h as in (**B**).

We have previously discovered that the honeybee product propolis (Manuka Health New Zealand Ltd, Te Awamutu, New Zealand) augments ERK signaling in HCT-116 cells, and this modulation has a pro-apoptotic effect in the cells [29]. Therefore, we reasoned that compared to hyperthermia alone, the combined exposure of CRC cells to hyperthermia and propolis may result in higher levels of ERK activation, and consequently, lower levels of clonal growth. To establish the effect of propolis on CRC cells under hyperthermia, we analyzed the major survival pathways via Western blot analyses (Figure 7A). In addition to inducing pERK1/2 at earlier time (*i.e.*, at 8 h of hyperthermia), propolis increased the levels of pc-JUN (used as a read-out for JNK activity) and suppressed pSTAT3 levels, as established previously [29,30]. Analyses of the clonal growth ability of HCT-116 and HKH2 cells revealed that both cell types decreased their clonal growth in a statistically significant manner when exposed to both hyperthermia and propolis. HCT-116 cells decreased their ability for clonal growth from 0.42% ± 0.11% when exposed to 42 °C to 0.009% ± 0.001% (*p* = 0.001) when exposed to both hyperthermia and propolis; HKH2 cells decreased their clonal growth from 1.73% ± 0.31% when exposed to 42 °C to 0.76% ± 0.33% (*p* = 0.004) when exposed to both hyperthermia and propolis (Figure 7B). This combined effect was not detected in normal human fetal colonic cells CCD841CoN. WNT/beta-catenin transcriptional assays with the two neoplastic cell types revealed that the effects of propolis are not due to modulation of WNT/beta-catenin signaling (Figure 7C).

## 3. Discussion

It has been postulated that the duration of ERK activity influences cell fate: Sustained activity leads to differentiation or apoptosis; whereas, transient pulse-like ERK signaling supports proliferation [31,32,33]. In agreement with the differential outcomes of transient and stable ERK signaling, HCT-116 cells that maintained increased pERK1/2 levels for up to 24 h at 42 °C (Figure 2) committed to high levels of apoptosis (Figure 1E); whereas, HKH2 cells that exhibited transient increase in pERK1/2 levels (Figure 2), were relatively resistant to hyperthermia-induced apoptosis (Figure 1E). Similar results were obtained in two addtional isogenic pairs of CRC cell lines; albeit, compared to HCT-116 cells, the mutant *KRAS* SW48 and DLD-1 cells were less sensitive to hyperthermia (Figure 2). The lower sensitivity to hyperthermia in DLD-1 cells is likely due to the mutation status of *TP53* [13,14]. In SW48 cells, the interpretation of how the *KRAS* mutation status modulates ERK1/2 activation and sensitivity to hyperthermia is confounded by the fact that the cells carry a hyperactivating mutation in *EGFR* [34]. The differential mutation status in *EGFR* may also explain why the anti-proliferative role of hyperthermia-induced ERK1/2 activity was observed at statistically signficant levels in mutant *KRAS* HCT-116 cells, but not in mutant *KRAS* SW48 cells (Figure 3).

In addition to sustained ERK activity under hyperthermia, mutant *KRAS* HCT-116 cells exhibited higher levels of WNT/beta-catenin transcriptional activity than wild type *KRAS* HKH2 cells (Figure 6A). This observation is in agreement with reports that oncogenic KRAS enhances WNT/beta-catenin transcriptional activity *in vivo* [28], and ERK activity augments WNT/beta-catenin signaling by increasing nuclear beta-catenin partly through downregulation of E-cadherin [35,36,37]. Alternative pathways, via which mutant tyrosine kinases and the downstream KRAS and ERK activity augment WNT/beta-catenin activity have been extensively researched [38], and yet, novel interaction levels between the two pathways are continuously discovered. For example, a recent report suggests that mutant KRAS may indirectly increase WNT/beta-catenin activity by suppressing the noncanonical WNT pathway [39]. Of relevance to our research focus, the positive crosstalk between tyrosine kinases/KRAS/ERK activity and the deregulated by mutations WNT/beta-catenin activity may result in levels of WNT/beta-catenin transcriptional activity that exceed the optimal for proliferation levels that are maintained by mutations (e.g., mutations in *APC, CTNNB1*, *etc.*), and such hyperinduction of WNT/beta-catenin signaling has been shown to trigger apoptosis in CRC cells [40,41]. Our results support the existence of a positive feedback between the two pathways in HCT-116 cells, the most sensitive to hyperthermia cells, since (a) the suppression of ERK1/2 activity decreases WNT/beta-catenin levels under hyperthermia (Figure 5A), and (b) the induction of WNT/beta-catenin activity with two different glycogen synthase kinase 3-beta inhibitors leads to increased pERK1/2 levels (Figure 5D and Figure 6B). However, SW48 cells with an activating mutation in *KRAS* did not replicate all of these observations, and we posit that this differential response is due to the fact that in these cells ERK activity is dependent not only on the mutant *KRAS* allele, but also on the mutant *EGFR* allele [34]. The *KRAS* mutant SW48 cells have been derived artificially, and it is unlikely that both mutations co-exist in cancer cells that have not been genetically manipulated. Analyses of The Cancer Genome Atlas colorectal adenocarcinoma data through the cBioPortal have revealed that mutations in *KRAS* and *EGFR* tend to be mutually exclusive (in total of 631 samples, http://www.cbioportal.org/, accessed on 13 April 2016).

**Figure 8:**
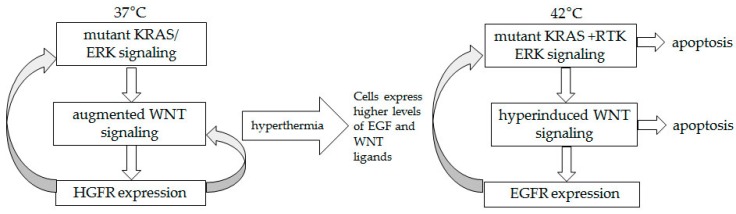
Proposed signaling amplifying mechanism in HCT-116 cells exposed to hyperthermia. At 37 °C, mutant *KRAS* supports ERK signaling, which in turn augments the already deregulated by a mutation WNT/beta-catenin activity. Signaling through HGFR, a transcriptional target of WNT/beta-catenin activity, feeds back positively the ERK and WNT pathways. At 42 °C, hyperthermia-induced EGF and WNT ligands allow for a switch from HGFR to EGFR signaling, and to increasing in time signaling amplifying mechanism.

The possibility of increased crosstalk resulting in augmented WNT and ERK signaling in HCT-116 cells at 42 °C could be explained in part by the ability of hyperthermia to induce high levels of cytokine expression, including that of EGF and canonical WNT ligands [42,43]. Interestingly, the receptor of EGF, EGFR, is a transcriptional target of WNT/beta-catenin signaling [30], and this may explain the increasing levels of total EGFR in HCT-116 cells under hyperthermia (Figure 4C). Phosphorylated EGFR levels also increased under hyperthermia in HCT-116 cells, and mirrored the levels of total EGFR (Figure 4C). The concomitantly increased phosphorylation of SOS1 in HCT-116 cells confirmed the involvement of autocrine RTK signaling under the conditions of hyperthermia (Figure 4A,B). Therefore, at 42 °C, HCT-116 cells sustained their pERK1/2 levels not only due to the *KRAS* mutation status, but also due to autocrine signaling through EGFR. In contrast, in wild type *KRAS* HKH2 cells, the autocrine signaling through EGFR was already induced at 37 °C, in absence of hyperthermia (compare phosphorylation levels of SOS1 and pEGFR levels, Figure 4A,D), and this may be a mechanism that allows the cells to compensate for the lack of *KRAS* mutations. When exposed to 42 °C, HKH2 cells were unable to induce in a sustained manner the levels of total or phosphorylated EGFR (Figure 4D). Based upon these observations, we posit that in cells with a mutant *KRAS* (*i.e.*, HCT-116) there is a positive feedback mechanism between ERK and WNT/beta-catenin signaling; whereas, in wild type *KRAS* cells (*i.e.*, HKH2), such mechanism is not functional as ERK1/2 activity is dependent only upon autocrine RTK signaling that is a subject to negative feedback mechanisms [31,32,33,34,35,36,37]. The positive feedback between WNT and ERK signaling has been discussed by others [38,39]; however, the mechanism has been studied under the physiological temperature of 37 °C and under such conditions it increases cancer cell survival. Here for the first time we demonstrate that under the conditions of hyperthermia, hyperactivation of both ERK and WNT activity contributes to increased cancer cell death. Manipulation of the two pathways (via pharmacological inhibitors) provided evidence for the role of each pathway in the suppression of CRC cell growth under hyperthermia. Based upon the data with HCT-116 cells, the most sensitive to hyperthermia cells in our studies, we propose the existence of an amplifying signaling mechanism between WNT/beta-catenin and ERK signaling under the conditions of hyperthermia (Figure 8), and this hypothetical crosstalk needs further validation.

Future studies need to explore the modulation of survival pathways induced by combination approaches involving hyperthermia (e.g., hyperthermia and propolis, or hyperthermia and pharmacological inducers of WNT/beta-catenin activity), and translate the *in vitro* findings to *in vivo* understanding of how hyperthermia impacts neoplastic cells. Understanding of the molecular mechanisms of hyperthermia-induced cancer cell death may drive the design of combination approaches that shorten the exposure to hyperthermia, and therefore, increase the safety of this anti-cancer treatment. In this aspect, our study has established that the combined exposure of CRC cells to hyperthermia and propolis increased pAKT and pcJUN levels, suppressed pSTAT3 levels, and sustained longer activation of ERK1/2 (Figure 7). Therefore, propolis augmented the cell growth inhibitory effect of hyperthermia (Figure 7B) not only through prolonged activation of ERK1/2, but also through inhibition of pSTAT3 levels (Figure 7A). Whether cell growth inhibition can be increased further by modulation of AKT and JNK signaling will be a focus of future studies; however, it is clear that propolis may counteract the anti-proliferative effect of hyperthermia by reducing WNT/beta-catenin transcriptional activity (Figure 7C). Confirming previous reports that normal cells are less sensitive to hyperthermia than neoplastic cells [8], the normal human fetal colonic cells CCD841CoN exhibited lower clonal growth at 37 °C and higher clonal growth at 42 °C, compared to the two neoplastic cell types HCT-116 and HKH2 (Figure 7B). This differential response to hyperthermia in neoplastic and normal cells could be exploited further in order to protect normal cells from any damage inflicted by hyperthermia.

Although discussed and researched for centuries, hyperthermia is seldom applied as a mainstream therapy or even as an adjuvant approach in the U.S. However, the recent development of immune and combination therapies has resurrected the interest in hyperthermia. For example, hyperthermic intraperitoneal chemotherapy (HIPEC) incorporates hyperthermia by heating the chemotherapeutic agents to 42 °C and infusing them into the peritoneal cavity of cancer patients after surgery. This approach is applied for CRC, appendix cancer, peritoneal mesothelioma, uterine cancer, and ovarian cancer. A number of U.S. hospitals are offering HIPEC as a treatment option outside or within clinical trials, and a search on http://www.clinicaltrials.gov website has revealed more than 80 trials that include HIPEC and more than 400 studies that apply hyperthermia for cancer treatment (as of February, 2016). Knowledge of how the cancer mutation profile impacts the response of patients to hyperthermia can greatly enhance the success rate of HIPEC and other anti-cancer therapies that incorporate hyperthermia. The lesson from the application of molecularly targeted therapies is that if we do not match the drug with the responsive mutation profiles, the outcomes are disappointing, and the therapies are erroneously deemed ineffective. The results from our study suggest that within the context of precision medicine, hyperthermia could be guided by the CRC mutation profile. Since HIPEC is applied with a variety of chemotherapeutic agents [44], these variations might also modify the hyperthermia sensitivity of cancer. Therefore, further investigation of these integrated approaches is required to establish the precise sensitivity pattern dictated by the combination of cancer mutations.

## 4. Experimental Section

### 4.1. Cell Culture and Chemicals

Human CRC cells HCT-116 and human fetal colonic cells CCD841CoN were obtained from the American Type Culture Collection (Rockville, MD, USA). The identity of HCT-116 cell line was confirmed by short tandem repeat analysis. HKH2 cells are isogenic to HCT-116 cells, and were derived by disruption of the mutant *KRAS* allele via homologous recombination [23]. The two additional isogenic pairs of CRC cells (DLD-1 and SW48 cells with and without a mutant *KRAS* allele) were bought from Horizon Discovery Group (Cambridge, UK). All cells were grown in alpha-MEM medium with 10% fetal bovine serum (FBS). Caffeic acid phenethyl ester (CAPE)––enhanced propolis (Propolis with Cyclopower) was a gift from Manuka Health New Zealand Ltd (Te Awamutu, New Zealand), AZD6244 was bought from Selleck Chemicals (Houston, TX, USA), TWS119––from Santa Cruz Biotechnology (Dallas, TX, USA), lithium chloride––from Sigma Aldrich (St. Louis, MO, USA), and FR 180204 from Tocris (Bristol, UK). All agents but lithium chloride were resuspended in dimethyl sulfoxide, and aliquots of the stock solutions were stored at −80 °C. Mock treatments included dimethyl sulfoxide in a volume equal to this of the treatments with agents. Lithium chloride was stored as an 8 M stock solution in water.

### 4.2. Western Blot Analyses, Antibodies, RTK Activation Analysis

Western blot analyses were performed as reported previously [45]. The following antibodies were used: anti-phospho-p44/42 MAPK (ERK1/2) (Thr202/Tyr204) (#4370, Cell Signaling Technology, Beverly, MA, USA), anti-phospho-STAT3 (Tyr705) (#9145, Cell Signaling Technology), anti-Ser473-phosphorylated AKT (sc-7985, Santa Cruz Biotechnology, anti-AKT (sc-8312, Santa Cruz Biotechnology), anti-beta-ACTIN (A5441, Sigma-Aldrich), anti-ERK1/2 (#9102, Cell Signaling Technology), anti-SOS1 (#07-337, Millipore, Billerica, MA, USA), anti-STAT3 (#9139, Cell Signaling Technology), anti-pcJUN, anti-c-JUN (sc-16312-R and sc-45, Santa Cruz Biotechnology), anti-pEGFR (pTyr1068, #3777, Cell Signaling Technology), anti-pHGFR (pTyr1234/1235, #3077, Cell Signaling Technology), anti-EGFR (#4267, Cell Signaling Technology) and anti-HGFR (#3127, Cell Signaling Technology), anti-beta-catenin (sc-53483, Santa Cruz Biotechnology) and anti-active beta-catenin antibody that recognizes the protein only when dephosphorylated on Ser37 or Thr41 (#05-665, Millipore). Antibodies to confirmed WNT/beta-catenin targets (FRA-1, c-JUN, MMP2, E-cadherin, and c-MYC) were from Santa Cruz Biotechnology. Antibodies were utilized at working dilutions of 1:1000. Total cell lysates were obtained via two different methods: by utilizing a sodium dodecyl sulfate -containing lysis buffer and quantifying the protein concentration [45], or by lysing equal number of cells directly in Laemmli buffer. Nuclear lysates were prepared with Nuclei EZ prep (NUC101, Sigma-Aldrich). Routinely, cells were plated 24 h prior to treatment. Activation of RTKs in the cells was performed after 16-hour incubations at 42 °C. We utilized a Proteome Profiler human phospho-RTK array (R&D Systems). Nitrocellulose membranes with spotted in duplicate capture antibodies were incubated with cell lysates. After binding the extracellular domains of phosphorylated and unphosphorylated RTKs, unbound material is washed away and a pan anti-phospho-tyrosine antibody conjugated to horseradish peroxidase was used to detect phosphorylated tyrosines on activated receptors by chemiluminescence.

### 4.3. Apoptotic and Clonal Growth Assays

For apoptotic assays, cells were plated 24 h prior to analyses in 24-well plates at 70,000 per well, and exposed to treatments for 24 h. All cells were harvested and stained with PE Annexin V Apoptosis Detection Kit I (#559763, BD Biosciences, San Jose, CA, USA). Flow cytometry was carried out with FACS Aria II and DiVa software; we analyzed total of 50,000 events per sample. Percent apoptosis is the number of apoptotic cells divided by the total number of analyzed cells and multiplied by 100. For clonal growth analyses, we plated 100 or 200 cells per well (for 37 °C exposure) and 1000–5000 cells per well (for 42 °C exposure) in a six-well plate and treated for 24 h. Colonies were counted at 14 to 20 days after the treatment). All experiments were performed four to six times with triplicate samples per experiment.

### 4.4. Transcriptional Assays

WNT/beta-catenin transcriptional activity was measured with the luciferase reporter pair TOP-Flash, containing a promoter with wild-type LEF/TCF binding sites, and FOP-Flash, containing a promoter with mutated (not functional) LEF/TCF binding sites. CRC cells were transfected transiently with the reporter vectors by the reverse Lipofectamine protocol. The vector pRSV-TK (Promega, Madison, WI, USA) was added to each transfection reaction for normalization of transfection efficiency. After transfection, cells were exposed to 37 °C or 42 °C for 16 h. Luciferase reporter assays were performed with a Turner Luminometer and a Dual Luciferase kit (Promega).

### 4.5. Statistics

All data were presented as mean ± standard deviation from at least three sets of independent experiments. Unpaired student *T*-test analysis was used to determine the significance of statistical differences. Differences were considered significant at *p* < 0.05.

## 5. Conclusions

We have shown that CRC cells that differ only in mutation status of *KRAS* exhibit differential sensitivity to hyperthermia. Compared to the relatively hyperthermia-sensitive mutant *KRAS* cells, wild type *KRAS* cells exhibit lower levels of ERK activation and lower levels of WNT/beta-catenin transcriptional activity under hyperthermia. Suppressed phosphorylation of ERK1/2 increases the survival of CRC cells exposed to hyperthermia; whereas, increased WNT/beta-catenin activity decreases cell survival under hyperthermia. These observations allowed us to hypothesize that the increase of the oncogenic ERK and WNT/beta-catenin activities leads to increased apoptosis and decreased ability for clonal growth under hyperthermia.
